# The Stereochemical Basis of the Genetic Code and the (Mostly) Autotrophic Origin of Life

**DOI:** 10.3390/life4041013

**Published:** 2014-12-16

**Authors:** Juan C. Fontecilla-Camps

**Affiliations:** 1 Metalloproteins Unit, University Grenoble Alpes, Institut de Biologie Structurale, F-38044 Grenoble, France; E-Mail: juan.fontecilla@ibs.fr; Tel.: +33-0-4-57428524; 2 Commissariat à l’Energie Atomique, Institut de Biologie Structurale, F-38044 Grenoble, France; 3 Centre National de la Recherche Scientifique, Institut de Biologie Structurale, F-38044 Grenoble, France

**Keywords:** primordial soup, stereochemical era, genetic code, autocatalytic cycle, low-specificity catalysts, origin of life

## Abstract

Spark-tube experiments and analysis of meteorite contents have led to the widespread notion that abiotic organic molecules were the first life components. However, there is a contradiction between the abundance of simple molecules, such as the amino acids glycine and alanine, observed in these studies, and the minimal functional complexity that even the least sophisticated living system should require. I will argue that although simple abiotic molecules must have primed proto-metabolic pathways, only Darwinian evolving systems could have generated life. This condition may have been initially fulfilled by both replicating RNAs and autocatalytic reaction chains, such as the reductive citric acid cycle. The interactions between nucleotides and biotic amino acids, which conferred new functionalities to the former, also resulted in the progressive stereochemical recognition of the latter by cognate anticodons. At this point only large enough amino acids would be recognized by the primordial RNA adaptors and could polymerize forming the first peptides. The gene duplication of RNA adaptors was a crucial event. By removing one of the anticodons from the acceptor stem the new RNA adaptor liberated itself from the stereochemical constraint and could be acylated by smaller amino acids. The emergence of messenger RNA and codon capture followed.

## 1. Introduction

Early propositions [1], followed by the original Miller-Urey [2] and later spark-tube experiments [3,4], along with meteorite content analyses [5,6], have been interpreted as evidence for a heterotrophic origin of life. According to this idea, commonly represented by the well-known “primordial soup” concept, abiotically generated amino acids, as well as other organic molecules, either synthesized on Earth or brought in by celestial bodies, were the first building blocks of life. According to the carbon-isotopic composition of apatite inclusions from the Isua belt in Greenland the first life on Earth appeared not later than about 3800 Myr ago, approximately 700 Myr after the planet’s formation [7]. Conversely, the earliest known microfossils, dated at 3500 Myr ago, are already structurally complex [7] and belong to microorganisms that were most likely modern in most aspects including a DNA-based genetic code. Although, as we will see below, views concerning the nature of an initial genetic coding or its absence are highly variable, it is generally agreed that the extensive involvement of RNA in contemporary protein synthesis strongly suggests it played a fundamental role in the very early stages of peptide formation. Consistent with this view RNAs are thought to have appeared very early on Earth both as self-replicators and catalysts [8]. Indeed, the discovery of ribozymes (catalytic RNAs), led W. Gilbert to propose the existence of an “RNA World”, predating the emergence of DNA and proteins [9]. The most appealing aspect of this concept is the plausible unification of informational capability and catalysis in the same molecule. Furthermore, these RNAs should have been capable of undergoing Darwinian evolution catalyzing many relevant reactions in a “RNA World” scenario [10].

## 2. Origin of the Genetic Code and the First Peptides

### 2.1. Theories about Codeless Peptide Synthesis and the Code Appearing before Translation

Some authors have proposed that the synthesis of the very first polypeptides took place in the absence of a code [11,12]. Thus, Krupkin *et al.* [12] have analyzed ribosome X-ray structures and proposed that the peptidyl transferase center (PTC) of the ribosome originally evolved to perform RNA World reactions unrelated to peptide synthesis and was subsequently recruited to catalyze non-coded amino acid polymerization [11,13]. Conversely, Johnson and Wang [14], who have analyzed amino acid side chain-ribonucleotide interactions in the ribosome, concluded that the genetic code may have developed in two stages: a first stage when prebiotically available amino acids formed peptides using a code that was not influenced by anticodon-amino acid interactions and a second stage when these interactions became essential and the code expanded. This latter stage has been defined by Yarus as the “stereochemical era” of genetic code evolution [15]. Rodin *et al.* [16] have published an exhaustive analysis of the stereochemical concept and concluded that the genetic code originated before translation. Along similar lines, several authors have proposed that RNAs could have bound (abiotic) amino acids to either improve stability or expand their functional capabilities, or both [11,17,18,19]. This interaction would have eventually led to the recognition of specific amino acids by RNAs predating the emergence of peptide synthesis [18] and initiating the above-mentioned “stereochemical era” of amino acid-anticodon interactions, which was not immediately related to code evolution. At this stage, (ribo)synthetase-adaptor complexes already carry anticodons that are recognized by cognate amino acids. The amino acid binds to this anticodon triplet and esterifies the adaptor, which then dissociates from the (ribo)synthetase. The modified RNA is postulated to be a proto-tRNA [20] or it uses the amino acid as a functional handle [18]. Alternatively, Wong has proposed that initial peptidation (or aminoacylation) of RNAs was not specific and that amino acid-codon (not anticodons) interactions appeared later in evolution [21].

### 2.2. Where Did the First Peptides’ Amino Acids Come from?

Regardless which hypothesis about the origin of early polypeptide synthesis is considered it is generally accepted in most of the relevant studies published so far that the first biologically polymerized amino acids were of abiotic origin. For instance, based on the concept of co-evolution of the genetic code with amino acid biosynthesis, Wong [21] has defined two types of amino acids depending on whether they were initially supplied by the environment (Phase 1) or were biosynthetically produced (Phase 2). This author classified Gly, Ala, Ser, Asp, Glu, Val, Leu, Ile, Pro and Thr as Phase 1 amino acids and Phe, Tyr, Arg, His, Trp, Asn, Gln, Lys, Cys and Met as Phase 2 amino acids. At some point during life evolution metabolic pathways developed so that biosynthetically produced Phase 1 amino acids replaced abiotic ones and Phase 2 amino acids emerged and were incorporated into proteins. At this point the genetic code expanded through co-evolution and codon capture by these new amino acids and eventually reached its present state [22]. Attractive as they may appear at first sight these ideas are not without conceptual difficulties because the use of abiotic amino acids in early functional peptides or in complex with RNAs is difficult to rationalize. If we consider spark-tube experiments [2,3,4] and analysis of meteorites [5] as valid models for the genesis of building blocks at life origins, then small simple amino acids, such as Gly and Ala -the latter as a racemic mixture- should have been by and large the most abundant in the primordial oceans and other possible early settings. However, binding of these small molecules to RNAs, as handles lacking large functional side chains, was unlikely to provide these nucleic acids with any significant evolutionary advantage [18]. The same reasoning applies to peptides mostly composed of these simple amino acids [21]. Conversely, more functionally relevant amino acids, such as Glu or Asp, must have been present in significantly lower concentrations in the soup [4] and unless one invokes some kind of early coding unrelated to the current one and restricted to a few amino acids [23], their covalent binding to RNAs and subsequent polymerization should have been seriously hindered by interference from, among others, the very abundant Ala and Gly. Another serious limitation to the functional diversity that these molecules could have conferred to RNAs and to early free polypeptides is the systematic absence of abiotically produced basic amino acids, such as Arg, Lys and His [24]. This absence would be especially problematic if these peptides were to interact with nucleic acids. A second source of concern is the biological retention of only a set of (l-) amino acids from large amounts of abiotic racemic mixtures of these molecules. Weber and Miller [25] have provided a rationale for the absence from proteins of some amino acids such as ornithine, which would undergo lactamization in peptides and upon acylation to RNA. However, the same approach does not work for others. For instance, α-amino-n-butyric acid, norvaline and norleucine are abiotically found in significant amounts although they are not proteogenic [25].

### 2.3. The “Stereochemical Era” of Code Evolution

As may be expected, small amino acids like Gly, Ala, Pro and Ser do not generate cognate RNA anticodons in SELEX experiments carried out to test the “stereochemical era” concept of amino acid-anticodon interactions [15]. Conversely, specific and highly statistically significant anticodon-amino acid interactions have been demonstrated for Arg, Ile, His, Phe, Tyr and Trp [15]. These results are consistent with those obtained by Johnson and Wong [14] in their ribosomal RNA-protein interaction study. These authors found that Gly, Ala, Val, Pro, Ser, Glu and Thr do not bind to RNA regions containing their anticodon sequences whereas the remaining ribosomal proteins amino acid side chains, except Asn and Cys, interact with RNA sequences corresponding to their anticodons (Table 1). As mentioned above, these observations led Johnson and Wong to conclude that the genetic code may have developed first independently from amino acid-anticodon interactions and then sterochemically dependent on them. This leaves us with two options: either (i) some kind of “frozen accident” generated anticodon triplets for the small abiotic amino acids, which were not stereochemically determined and this process was followed and completed by stereochemically-assigned anticodons *of essentially the same nature* for the remaining larger amino acids; or (ii) a different primordial coding for small amino acids eventually evolved into the current triplet code with the subsequent stereochemically-determined incorporation of larger amino acids. Neither option seems especially plausible because they require either an improbable teleonomic event (the accidental emergence of a coding triplet with *future* stereochemical applications) or a very difficult to picture transition between different coding systems. Rather, the most parsimonious interpretation of these results implies that the first amino acids to be incorporated into early polypeptides *already* used a stereochemically-determined code and were for the most part the proto-metabolically generated, bulkier ones (although transcription errors were probably frequent and occasionally non-coded amino acid could have been incorporated) (Table 1). The contradiction between an initial stereochemically-determined code and the generally accepted idea that simpler amino acids were the first to be part of peptides and to enter the (modern) genetic code has already been addressed by Yarus and co-workers [15,26]. These authors have argued that RNA-based metabolic pathways could have generated the larger amino acids before the emergence of the code. Yarus also seems to disagree with the involvement of small abiotically generated amino acids as building blocks in early polypeptide synthesis [15]. Additional support for a stereochemical origin of the genetic code is the observed chirality of some biological amino acids. Indeed, a preference for l-Ile, l-Leu and l-Tyr over their d- counterparts has been observed in RNA aptamer binding experiments [15]. It is difficult to imagine a preference for l-amino acids in proteins arising from a “frozen accident” occurring in abiotic racemic mixtures of small amino acids.

**Table 1:** Comparison of abiotically synthesized amino acids (Miller-Urey, [2]) and amino acids that interact with their anticodons in the ribosome (Johnson-Wang, [14]).

Amino Acid	Miller-Urey	Johnson-Wang
A	+	
C		N/D
D	+	+
E	+	
F *		+
G	+	
H *		+
I *	+	+
K		+
L *	+	+
M		+
N		
P	+	
Q		+
R *		+
S	+	
T	+	
V	+	
W *		+
Y *		+

Note: * Amino acids that bind anticodons in SELEX experiments [15].

## 3. Discussion

### 3.1. Abiotic Amino Acids as unlikely Components of the First Peptides

**Figure 1:**
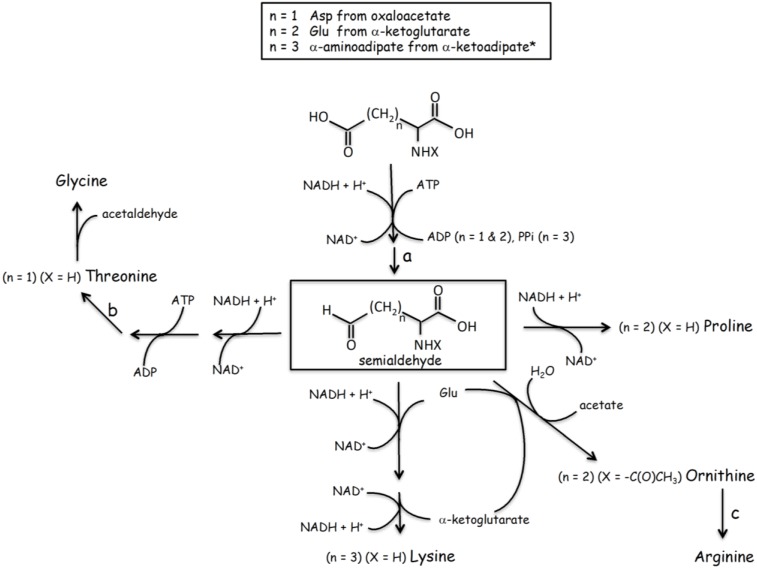
“Tricarboxylic acid cycle-semialdehyde” route for Pro, Thr, Lys and Arg synthesis. a = dephosphorylation; b = PLP-dependent dephosphorylation; c = urea cycle. The starting substrates are aminated versions of intermediates of the pathways in Figure 2.

Based on the arguments outlined above, I will argue that the concept of simple abiotic amino acids being the first components of early polypeptides is likely to be incorrect. In the “primordial soup” concept it is assumed that the first abiotic amino acid synthetic pathways were later replaced by proto-biosynthetic ones [27], an obvious requirement for life to become independent of highly contingent external factors. However, as it might be expected by the very different nature of the reaction conditions, there is no clear link between abiotic amino acid production (and abundance) and the corresponding extant biological synthetic pathways. Conversely, examination of extant metabolisms shows a clear correlation in terms of the catalyzed reactions between synthetic pathways of chemically very different amino acids [28]. As a proof of concept I will use here what I will call the “tricarboxylic acid cycle (TCA)-semialdehyde” route for amino acid synthesis (Figure 1). Semialdehydes obtained from carboxylated amino acids are intermediates in the synthesis of Pro, Thr, Lys and Arg and are formed through a set of closely related reactions including phosphorylations (ATP), hydride-based reductions (NAD(P)H) and, in the cases of Lys and Arg, transaminations (Glu). A common set of primitive catalysts with rather low and broad specificity could have catalyzed these reactions in early settings. Such commonality would have allowed for several related metabolic pathways to co-exist in a given environment using a shared set of catalysts. Although Pro and Thr may not have been initially recognize by RNA anticodons they would have been readily available when the genetic code expanded (see below).

**Figure 2:**
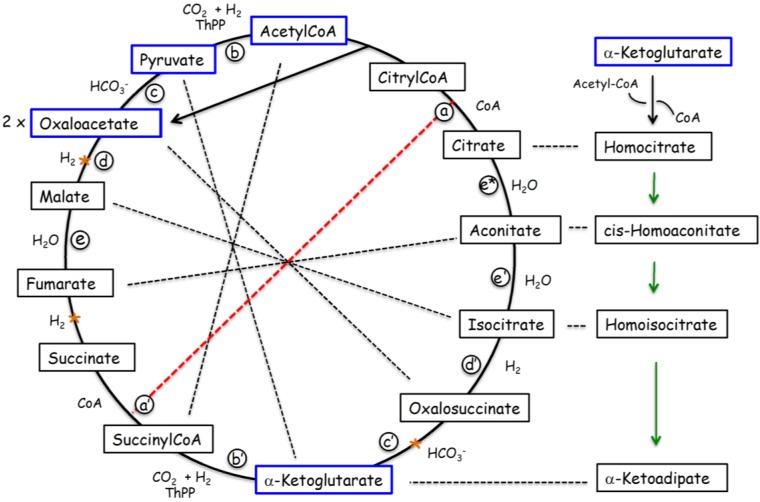
Tricarboxylic acid (TCA) cycle (blue frames depict central metabolites)*.* In its reductive version (counterclockwise in the figure, [29]), carbon is fixed through the generation of two oxaloacetate molecules per cycle. One of these molecules is directly produced from citrylCoA (black arrow) and the other one through acetylCoA and pyruvate (circle). The TCA cycle can be divided into two halves (red dashed line) with approximately equivalent substrates (connected by black dashed lines) and reactions (indicated by a–d and a′–d′; e′ = e *). Similar substrates and reactions are also used for the synthesis of α-ketoadipate (right side of the figure; see also Figure 1) and α-ketoisocaproate (not shown,) precursors of lysine and leucine, respectively.

The existence of a common set of primitive catalysts is also supported by the similarity in terms of substrates and enzymes between two halves of the TCA [29] (Figure 2) and between these halves and pathways corresponding to the synthesis of α-ketoadipate from α-ketoglutarate (Figure 1) and of α-ketoisocaproate from α-ketoisovalerate, precursors in lysine and leucine syntheses, respectively. These catalysts could have successively been inorganic mineral surfaces or globules [30,31], organic cofactors such as nucleotides [32], ribozymes [33] and finally enzymes. In its reductive version, the TCA cycle is a good candidate for an autocatalytic anabolic carbon-fixing pathway that could have operated in a pre-biotic setting [34,35,36]. Its importance in contemporary reactions is underscored by the central metabolic precursors that derive from it (Figure 2).

**Figure 3:**
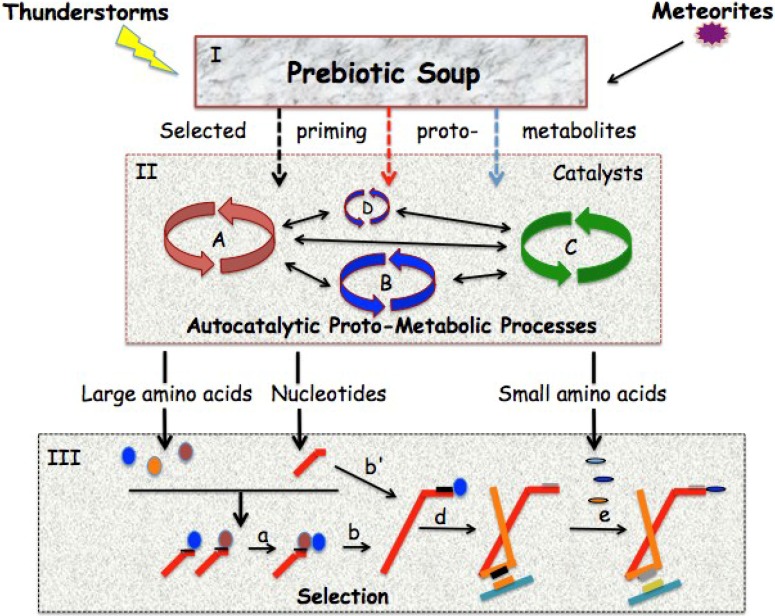
Proposed sequence for the origin of coded polypeptides. (**I)** Organic molecules resulting from atmospheric and geological phenomena and meteorite and comet showers may have accumulated in some primordial Earth settings. However, the resulting mixtures were most likely too heterogeneous to allow for the emergence of proto-biological processes. Instead, it is proposed here that some organic molecules were selected, either through thermal or pH gradients and/or diffusion, or through some other uncharacterized process, to penetrate a different compartment (II) where primordial catalysts were available; (**II**) Some of the organic molecules reaching this compartment are proposed to have primed several interacting autocatalytic cycles (A–D), which would have been involved in essential processes such as carbon fixation. A central idea of the hypothesis put forward here is that the initial compartment had a set of primitive catalysts with low specificity capable of catalyzing similar reactions with different but related substrates (see Figure 1 and Figure 2). Another important notion is that this catalyst set could progressively evolve, for instance from minerals to nucleotides to peptides, still catalyzing the same or closely related reactions, without disrupting the functioning proto-metabolic chains. Thus, some products of the evolving metabolic pathways could have become, in a virtuous cycle, catalysts themselves, ever improving the efficiency and the selectivity of the reactions; (**III**) This compartment could be the same as (II) or be connected to it in an intimate way. Here, in the presence of many other molecules such as sugars, carboxylic acids and amines, large amino acids (colored ellipsoids) could have interacted with nucleotides (red l-shaped bars). These interactions would have eventually led to the emergence of anticodon-like structures in the nucleotides (black thin bars) destined to improve their recognition and binding of amino acids functionally important in “RNA World”-type reactions. Eventually, interactions between amino acid-loaded nucleotides could have led to the formation of oligopeptides (reaction a) in a manner similar to the reaction catalyzed by the ribosome. Formation of oligonucleotides (reactions b and b′), which may have improved ribozymes’ catalytic properties led to larger molecules still capable of carrying large functional amino acids and synthesizing oligopeptides containing them (not shown). The crucial gene duplication event, a central component of the hypothesis advanced here, (reaction d) positioned one of the anticodons away from the arm carrying the amino acid thus allowing its evolution towards the recognition of small amino acids (small colored ellipsoids; reaction e with transition from a black to a gray thin bar representing anticodon evolution). These amino acids could now also be incorporated into oligopeptides (not shown) Proto-messenger RNA (green bars) most likely appeared at this time evolving their codons (orange and yellow thin bars) to match the expansion of the anticodon repertoire including now the recognition of the small amino acid set.

Although it is argued here that abiotic amino acids were not included in the first polypeptides, other abiotically produced molecules, such as pyruvate and citrate in the case of the TCA cycle, could have primed primitive versions of various metabolic pathways [37,38]. Thus, the combination of self-replicating, catalytic RNAs, the origin of which is not discussed here, with a continuous source of amino acids and other precursors from the reductive TCA cycle and other primordial pathways might have been all it took to get life started (Figure 3).

### 3.2. Gene Duplication of Primordial RNA Adaptors and Expansion of the Genetic Code

I will now address the problem of how I think, if the first anticodons were most likely stereochemically generated only for the bulkier amino acids, eventually simpler ones were also assigned their own anticodons/codons. In order to understand this process, we need to review the current views on tRNA evolution. It is generally accepted that modern tRNAs arose from ancestors that underwent a gene duplication event [39,40]. As early as 1978, a few years after the three-dimensional structure of Phe-tRNA became available [41], Hopfield [42] already speculated that the tRNA anticodon region had been contiguous to the acceptor stem before being displaced to the other side of the molecule by gene duplication. As discussed above, the idea of having the amino acid bound to its cognate RNA anticodon, next to where the amino acylation should occur, makes sense because it would protect the aminoacyl linkage against hydrolysis [42,43]. Two aminoacyl-RNAs could have then interacted to form a dipeptide [44] not unlike contemporary acylated tRNAs do in the peptidyl transferase site of the ribosome [45]. However, direct amino acid recognition by the adaptor could only be effectual if its side chain was bulky enough to interact with the cognate bases of a possible anticodon; this proto-tRNA molecule, like a vinyl music record, was an “analog” device. By moving one of the two resulting anticodons to the other end of the adaptor molecule the gene duplication event liberated the coding process from this restrictive anticodon-amino acid direct recognition. RNA aminoacylation must have also become progressively independent of direct amino acid recognition at the acceptor stem and more dependent on anticodon-codon recognition at the anticodon region (Figure 3). At this point, the tRNA molecule became, like a Compact Disc, a “digital” device. Schimmel *et al.* [46] have argued that there is an operational RNA code embedded in the acceptor stems of contemporary tRNAs. Although this code is now very different from the corresponding amino acid anticodons Rodin *et al.* [16] have postulated that they have a common origin.

The structural revolution represented by the gene duplication of the proto-tRNA, allowed for the emergence of non stereochemically-dependent but structurally similar anticodons for the smaller amino acids. Because anticodons could now interact with cognate codons, a coding messenger RNA molecule could evolve in a way perhaps akin to the one discussed by Yarus for “Direct RNA Templates” (Figure 3) [15]. Alternatively, adaptor, messenger and proto-ribosome activities could have initially been united in one RNA molecule. The possible existence of a multifunctional ancestral RNA is supported by several observations: (i) tRNA and rRNA share significant nucleotide sequences, scattered throughout the structures [47,48]; (ii) tRNAs have a nucleotide triplets pattern reminiscent of a gene-like coding sequence, which suggests that at one point adaptor and coding functions were united in the same RNA molecule [49] and (iii) an *in vitro* selected ribozyme displays both aminoacyl-RNA and peptidyl-RNA synthetic activity [50]. From the data discussed above it may be postulated that independent mRNA and tRNA molecules and protein-based acylating activity appeared later in evolution. Once a coding relationship was established between RNAs and peptides, some of the latter may have evolved into proto-enzymes under the selective pressure exerted to enhance the synthesis of their own constituting essential amino acids and increase the chances of making more RNA-coded polypeptides. Early polypeptides were probably short, of relatively simple composition and lacked well-defined three-dimensional structures. This notion is supported by the crystal structure of the ribosome the proteins of which primarily interact with the RNA catalytic core through their extended unfolded segments [51]. Eventually, enzymatic takeover of most metabolisms, through gene duplication and refinement of the protein synthetic machinery, must have taken place.

## 4. Conclusions

Life may have first appeared in rather confined settings with the contribution from abiotic sources mostly limited to the initial precursors of nucleotides and pre-metabolic pathways such as the TCA cycle. A very early requisite for Darwinian-type evolution to occur is the persistence in time and space of molecule types and their reactions. This is what makes the “RNA World” concept so attractive. However, a similar restriction applies to pre- or proto-metabolic processes because they also need to evolve. This, I think, is the major problem with the “primordial soup” hypothesis because it lacks a plausible way of providing a continuous link between the use of abiotic amino acids in early peptides and the emergence of the corresponding biosynthetic pathways. Conversely, the connection between the “RNA World” and pre- or proto-metabolic pathways makes such link possible: if the very first amino acids interacting with RNA were already synthesized through a series of rudimentarily reproducible catalyzed reactions, evolution of these pathways became possible. Without introducing any radical changes in the nature of the reactions themselves catalysts could evolve from minerals to proto-enzymes while keeping a relatively broad specificity. The similar nature of many contemporary amino acid synthetic reaction pathways, like the ones outlined above, reveals their very probable common origin in a promiscuous environment where the same catalysts were involved in several reactions with similar substrates. Besides providing a plausible link between the RNA World and the first proto-metabolic pathways the notion that the first amino acids were synthetized through a series of rudimentarily catalyzed reactions makes sense within the concept of “stereochemical era” of genetic code evolution. This is so because peptides did not have to be mostly composed of the simplest amino acids, as the “primordial soup” hypothesis will imply, but of those that could be synthesized from molecules produced by proto-metabolic pathways such as the TCA cycle, including functionally relevant ones like aspartate and lysine.

Rather than postulating that the “stereochemical era” of genetic code evolution followed some other differently-based process of code generation or no code at all, it is proposed here that stereochemistry was the original determining factor of code evolution and of the chirality of proteogenic amino acids. Because, if this hypothesis is correct, only side chains large enough to generate a cognate anticodon could have been incorporated into early peptides, smaller abiotically produced amino acids probably were not significantly involved in this process. The gene duplication event that most likely affected the proto-tRNA liberated the coding process from the stereochemical constraint that the amino acid-anticodon recognition represented and allowed for its evolution through the incorporation of new amino acids through stereochemically independent but functionally related codon capture.
